# Digital competence of teachers in the use of ICT for research work: development of an instrument from a PLS-SEM approach

**DOI:** 10.1007/s10639-023-11895-2

**Published:** 2023-05-15

**Authors:** Francisco D. Guillén-Gámez, Julio Ruiz-Palmero, Melchor Gómez García

**Affiliations:** 1 Department of Pedagogy, Faculty of Teacher Training and Education, Autonomous University of Madrid (UAM), Madrid, Spain; 2 Department of Didactics and School Organization, Faculty of Education Sciences, University of Malaga (UMA), Malaga, Spain

**Keywords:** Digital competence, Technology, Research process, Teachers, Higher education

## Abstract

All spheres of our life are being affected using technology, particularly its integration in the research processes carried out by teachers. The success of the integration of specific digital resources in research work can be affected by several factors, such as: digital skills for finding information, managing it, analyzing it, and communicating results; digital flow; anxiety in the use of ICT; digital ethics; quality of digital resources; and finally, the behavioral intention to integrate ICT. The purpose of this study is to examine the factors that influence the integration of ICT in the research process of the Higher Education teacher, and the relation between them. An online survey was used to collect data, and 1740 participants. This study used a causal model through partial least squares structural equations modeling (PLS-SEM). With this, the hypotheses established between the integration of ICT and its possible incident factors were verified. The findings revealed a significant influence path from factor integration to digital skills, ethics, flow digital, and behavior intention. Although, resource quality and ICT anxiety had significant effects on the causal model, they did not have a large impact on teachers’ integration of digital resources. The total of these factors corresponded to 48.20% of the variance in the integration of the researcher of the specific digital resources to be used in the research process. These results confirm that this model is effective in explaining the technological integration of teachers to use ICT in research work.

## Introduction and problem statement

The current global pandemic that has appeared in 21st century society, caused by COVID-19, demands profound and revolutionary transformations that affect the symbiosis between the great deployment of existing technological devices on the market and the progress of science (Virgili, 2021). In this sense, in the context of Higher Education, a deep reflection is required on the part of teaching staff regarding how to systematise research processes in the digital era (López-Martín et al., 2017; Mandal, 2018) in order to be able to respond to this symbiosis.

Rubio et al. (2018, p. 336) states that “research competence in teachers of different disciplines contributes to social development and to the improvement of innovation and competitiveness of institutions”. But to achieve this end, it is necessary for Higher Education teachers to develop skills to formulate problems, pose hypotheses, experiment, analyse, interpret and communicate the results (Roth & Roychoudhury, 1993) and, above all, to develop a hybrid and comprehensive competence in both scientific and digital competences, through the use of new information and communication technologies (ICT) (George & Salado, 2019; Suárez-Triana et al., 2020). In other words, today’s society demands teachers who know how to respond to the challenges presented by an increasingly complex and changing reality (Gómez & Granados, 2013), with an increasingly positive attitude towards ICT, so that they constitute the tools of transformation (Semerci & Aydin, 2018). This challenge implies ongoing teacher training in scientific competences (Lovat et al., 1995), under the umbrella of efficiency in ICT integration (Tanjung, 2019; Guillén-Gámez & Ramos, 2021; Şimşek & Ateş, 2022), focused on generating scientific knowledge more quickly and effectively among members of the scientific community (Arcila-Calderón et al., 2015).

In recent years, different institutions and research groups have been reformulating and developing the concept of digital competence in teaching, attempting to delimit and qualify its dimensions (Ortega-Rodríguez et al., 2022). In the European context, the DigCompEdu (Digital Competence Framework for Educators) model has gradually been refined with the development of instruments such as, for example, the one created by Ghomi and Redecker (2019) which have been analyzed in multitus of educational scenarios. The TPACK model by Koehler and Mishra (2009) or the PEAT model which is still being developed under the framework of the Erasmus + Project “Developing ICT in teacher education” (DiCTE, 2019) has also been implemented with great acceptance. However, when examining the scientific literature on digital competence in higher education, there are still few studies that focus on the construction of instruments that measure digital competence in research work (Guillén-Gámez & Mayorga-Fernández, 2020; Martínez et al., 2020), and/or on how teachers use digital resources to search, analyse and communicate the results of their studies (Sim & Stein, 2016; Seraji et al., 2017; Robelo & Bucheli, 2018; Guillén-Gámez et al., 2020).

In an attempt to reverse this trend, this study aims to understand the different factors that influence the development of digital competence in research work. Therefore, the objective of this study has been to design and analyze the psychometric properties of an instrument which evaluates through a causal model those possible factors involved in the acquisition of digital competence of Higher Education teachers when they use digital resources in investigative work.

## Theoretical framework

Next, a conceptual approximation of the factors that affect the digital competence of teachers is carried out, as well as the incidence that some factors have on others. In addition, once the factors have been specified, the causal model is shown, which will be the basis for the creation of the instrument.

### Anxiety towards ICT

attitude is an outwardly manifested state of mind. Some authors have classified attitudes towards ICT in terms of anxiety or stress (Loyd & Gressard, 1984; Yildirim, 2000; Téllez et al., 2022), understood as a person’s level of reluctance or negative emotional state when having to integrate ICT into their professional duties (Simonson et al., 1987).

The literature affirms that certain attitudinal behaviours may be predictive of other future behaviours (Henerson et al., 1987; Babie et al., 2016; Knezek & Christensen, 2016; Ünal et al., 2019) stated that a teacher’s attitude influences the intention to use ICT, and consequently, its integration into professional tasks (Joo et al., 2018; Paraskeva et al., 2008).

### Digital Flow

the concept of flow state was first proposed by Csikszentmihalyi (1975) and defined as the combination of enjoyment and intrinsic interest in an activity, such that the experience intensifies with increased concentration on the task (Huang & Liao, 2017). Regarding ICT, Hoffman and Novak (1996) state that the more people that are immersed in a state of flow, the higher their expectations regarding future intentions to use them (Ahmad & Abdulkarim, 2019) and, consequently, the higher the actual use of technology (Kim & Jang, 2015). This finding is consistent with the conclusions of some research on flow experience with ICT (Calvo-Porral et al., 2017; Rodriguez-Sanchez et al., 2008). In addition, evidence was found to support the relationship between flow state and technological skills (Catino, 2000; Giasiranis & Sofos, 2017).

### Digital skills for finding information, managing it, analysing it and communicating results

research skills can be defined as the practical domain that a person has to go in search of a problem and its solution through the scientific method (Pérez & López, 1999) using ICT in this process (Hassani, 2015; Murnane & Levy 1996), in such a way that allows them to search for information, manage data and know how to communicate them (García et al., 2018). ICT skills is a key factor that will influence the integration of ICT use (Alazam et al., 2013; Teo, 2009), which could lead to a decrease in negative feelings (anxiety) towards ICT use (Revilla et al., 2017).

*Digital ethics: e*thics refers to the codes and norms that value human behaviour in a community (Dewey, 2008). Currently, the scientific community is facing a great ethical challenge in its research approach (Luke, 2018) as the so-called digital culture predominates. In this sense, it is considered that a good researcher should have adequate knowledge of the basic ethical principles of research (Sanjuanelo et al., 2007), employing good practices with ICT (Dominighini & Cataldi, 2017; Stahl et al., 2014). Since as evidenced by Stahl et al. (2017) having a good ethical awareness can contribute to the deployment of innovative practices with ICT.

*Intention and Integration: several* ICT studies have demonstrated the importance of an individual’s intentions in predicting integration behaviour (Anderson & Maninger, 2007; Venkatesh et al., 2003; Shiue, 2007, p. 427) assumes that “the extent of actual use is based on the teacher’s intention to use instructional technology”. The authors analyzed the intention to use 242 science teachers from Taiwan on the integration of ICT in the educational process, evidencing that “the intention to use instructional technology has the largest direct effect on its actual use” (p. 446). However, as stated by Banas and York (2014, p. 730) “while intention does not guarantee future behaviour, well-grounded research has established it as a reliable predictor”. For instance, in Czerniak et al. (1999) study, teachers’ intentions to use ICT predicted between 18% and 24% of the true variance in actual technology use. In the same context, the stronger a person’s intention to use an ICT resource, the more likely their integration will materialise (Olugbara & Letseka, 2020). However, teachers are reluctant to integrate technology as a teaching tool if the technology is not good (Shiue, 2007), so it is also necessary to consider the quality of digital resources in the teacher’s research process.

*Quality of ICT resources: a* variety of external factors have also been identified that can significantly influence the integration of ICT resources in teachers’ work, such as Internet accessibility (Lin et al., 2012), the software or hardware available in schools (Gil-Flores et al., 2017) or the lack of technical and training support (Lawrence & Tar, 2018).

### Research model

**Fig. 1:**
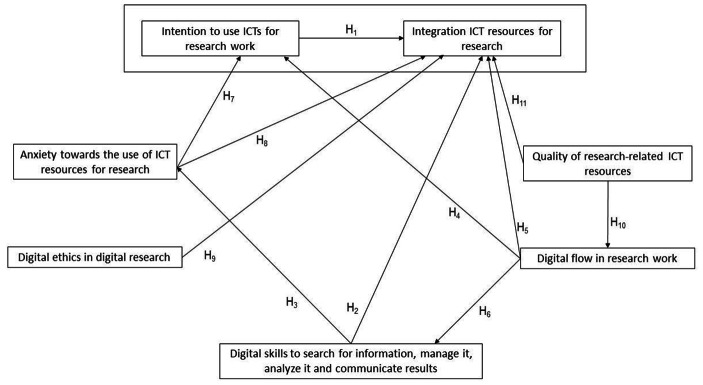
*Hypotheses of the proposed model*

in the present study, the causal theories analysed in the literature review are operationalised with the underlying factors and relationships, all shown in Fig. 1. Each arrow in the figure represents a hypothesis of the study, and from them we aim to analyse the viability of the structural equation model proposed, so that it explains the maximum proportion of variance shared between the exogenous and endogenous variables.

## Method

*Design and Participants.* A non-experimental quantitative survey-type methodology was used. The study population is made up of 122,910 Higher Education teachers from the Spanish Education System (MECD, 2019–2020). A non-probabilistic purposive sampling was used, collecting a total of 24,565 emails through the websites of the educational institutions, and subsequently contacting all of them by the same means. The sample consisted of 1740 teachers where 43.60% (n = 759) were female with a mean age of 48.15 ± 9.57years, while 56.40% (n = 981) were male teachers, with a mean age of 49.61 ± 29.53. Specifically, the participants belonged to the following areas of knowledge: Experimental Sciences (n = 248), Life Sciences (n = 206), Medical and Health Sciences (n = 261), Engineering and Architecture (n = 176), Social Sciences (n = 357), Legal Sciences (n = 118), Economic and Business Sciences (n = 165), and Humanities (n = 209).

*Ethical considerations.* Before the teachers filled in the online questionnaire, they have been informed about the purpose of the study. The data collection was carried out anonymously through a form without any label which could compromise the identity of the participants. During the data collection and in the presentation of the results in this study, the identity and confidentiality of the teachers has been kept private, guaranteeing the anonymity of the responses.

*Instrument.* In this study an instrument is created for the analysis of teachers’ digital competency, that enables the largest percentage of true variance in the integration of the use of ICT in research works to be explained, from a series endogenous and exogenous factors. The first version of the instrument was created by the authors of this study. In this version, the construct was operationally defined after a thorough review of the most used and relevant ICT resources, focusing on the interest in the predominant theoretical dimensions in scientific literature. Following this, it was reviewed by three experts in educational technology (validity of content). The adaption and relevance of the items and their underlying factors were evaluated, as well as the agreed review of the items. Eliminating those items with values less than 50% agreement between the experts.

As such, the original instrument remained composed of 40 questions made up closed categories. Each factor was measured on a seven-point Likert scale as follows: DIM.1 Digital abilities in searching for information, managing it, analysing it and communicating the results, from value 1 (I am notable to) to value 7 (I am able to); DIM.2 Digital ethics in digital investigation, from value 1 (I never do it) to value 7 (I do it frequently); DIM.3 Digital flow in investigation work, from value 1 (Totally disagree) to value 7 (Totally agree); DIM. 4 Anxiety towards the use of ICT in investigating, from value 1 (Totally disagree) to value 7 (Totally agree); DIM.5 Quality of ICT resources for investigation, from value 1 (It is poor) to value 7 (It is excellent); DIM. 6 Intention to use ICT resources for investigation work, from value 1 (Totally disagree) to value 7 (Totally agree); and DIM. 7 Integration ICT resources for research, from value 1 (I never do it) to value 7 (I do it frequently). Table 1 shows the items of each dimension together with their corresponding code.

**Table 1:** *Initial instrument*

DIM.	Code	Description
DIM. 1. Digital skills to search for information, manage it, analyze it and communicate results	D1_1	I know how to use software for the analysis of qualitative data (Atlas.ti, Nvivo, Ethnograph, Hyperresearch, Maxqda, QDA MINER, NUD*IST)
	D1_2	I know how to use audio and video editors to create and edit collected information through interviews, focal groups, etc. (Adobe Premiere, iMovie, Windows Movie Maker, Audacity)
	D1_3	I have abilities necessary for analysing quantitative data (SPSS, EXCEL, JAMOVI, AMOS, R, Minitab)
	D1_4	I know how to search in scientific data bases (ScienceDirect, ProQuest, PsycINFO, Redalyc.org, Scielo, Academia.edu…)
	D1_5	I know how to use Boolean operators (AND, NOT, OR, XOR) to refine my searches for scientific articles.
	D1_6	I have the skills to use bibliographical managers (Mendeley Zotero Endnote, Refworks) those which allow me to store bibliographic references and use such references in my studies following different citation rules.
	D1_7	I have abilities in managing my scientific social media, add my published studies and/or consult their reading statistics
	D1_8	I usually use scientific social media to interact with other investigators.
DIM. 2. Digital ethics in digital research	D2_9	I apply the rules of copyright when I share the results of my studies through scientific social media.
	D2_10	Before sending a study for its’ publication, I digitally check it and apply the publication rules employed in every editorial/journal (APA v.7; Chicago, Harvard…)
	D2_11	I check the original source, and the results of a study referenced by other authors in their original publications.
	D2_12	I check that the bibliography selected for my study comes from journals with a certain grade of scientific prestige (for example, that they use paired revision “double look”)
	D2_13	I check that in my studies there is no self-plagiarism or plagiarism of other studies.
DIM. 3. Digital flow in research work	D3_26	I find it gratifying to use ICT resources in my investigation works
	D3_27	I find it enjoyable to use software for the analysis of data both quantitative (SPSS, JAMOVI, R…) and qualitative, Atlas.ti, Nvivo…) to complete my research.
	D3_28	I am motivated by the thought that by using digital software for data design and analysis I can more easily publish my scientific achievements in high-impact journals.
	D3_29	I like to learn new digital resources that are going to allow me to analyse data and/or communicate the results in some software afterwards.
DIM. 4. Anxiety towards the use of ICT resources for research	D4_30	*It overwhelms me to think that I have to learn to use digital resources to collect data and analyse it with some software afterwards
	D4_31	*It makes me anxious to have to be constantly checking the impact indexes of the journals for if the quartile has increased or decreased.
	D4_32	* I get tired of having to constantly use ICTs to position and share my scientific publications and improve my digital reputation through the h-index and/or the i-index10.
	D4_33	* I get nervous when I have to teach a colleague and/or student some ICT resource related to research (Mendeley, SPSS, AMOS, Google form, Atlas.ti…).
	D4_35	*In general, I would prefer not to have to learn or use ICT resources for my research.
DIM. 5. Quality of research-related ICT resources	D5_22	My place of work had a good internet connection
	D5_23	My department or my investigation group buys ICT resource licenses that require an additional page
	D5_24	Mi department or my investigation group provides me with all the ICT resources I require for my investigations
	D5_25	My department or investigation group has strong devices (pc/laptops) available so that the technological resources function smoothly and quickly
DIM. 6. Intention to use ICTs for research work	D6_35	Assuming my educational institution provides me with ICT resources for research work, I intend to use them at some point in time.
	D6_36	If the institution to which I belong does not provide me with a certain ICT resource that I require for my research, I am responsible for obtaining it.
	D6_37	In the near future, I plan to continue learning how to use ICT resources to expand my research work.
	D6_38	I intend to further develop my training in the use of online scientific databases for my research.
	D6_39	I intend to continue to use and/or use bibliographic managers for my future studies.
	D6_40	I want to improve my use of social networks to transfer my research and interact with other researchers.
DIM. 7. Integration ICT resources for research	D7_14	I use anti-plagiarism programs (Plagium, Viper, Article checker, Turnitin, Compilatio, etc.)
	D7_15	I use bibliographic managers
	D7_16	I use social media to circulate my scientific publications
	D7_17	I use scientific data bases for access to read other studies
	D7_18	I use web search engines to consult bibliographies (Google academic / Google scholar)
	D7_19	I use videoconference systems to have meetings with my investigation group
	D7_20	I use Google + collaboratives to host my research data
	D7_21	I use data analysis programs (be it quantitative and/or qualitative)

Note: Items with * in their name have an inverse score

### Data analysis procedure and techniques.

This study used the partial least squared method (PLS) based on the analysis of principle components. This method falls within the family of structural equation models, where it is possible to carry out both the measurement model (reliability and validity of the underlying factor measurements) and the structural model (coincidence relations of the hypothesis established between the factors). For this purpose, the SmartPLS software was used, following the steps below:

1. Internal consistency of the instrument. For this, the Cronbach’s alpha coefficient was calculated, the Composite Reliability (CR), and the load factors of the items, with values greater than 0.707 (Carmines & Zeller, 1979).
2. Convergent validity. The average variance extracted (AVE) was obtained where values greater than 0.50 would indicate a good fit of the model (Bagozzi & Yi, 1988).
3. Discriminant validity. For this, three types of analysis were used. The criteria of Fornell-Larcker proposes that discriminant value exists between two underlying variables if the square root of the AVE coefficient of an underlying factor is greater than the variance of such factor together with the rest of the instruments’ factors. (Henseler et al., 2015; Clark & Watson, 1995) evaluate the discriminate value between underlying factors by means of heterotrait-monotrait correlations (HTMT), where a threshold less than 0.85 would be adequate. Lastly, the cross-loading analysis evaluates the grade in which an underlying variable is different from the rest of the variables, and consequently, their corresponding items measure that of the construct into which it has been incorporated.
4. Evaluation of the structural model. The quality is evaluated through the determination coefficient R^2^ which measures the amount of variance in the underlying endogenous variables (intention, integration, abilities, anxiety and digital flow) explained by the underlying exogenous variables (digital ethics and quality of resources). These criteria can be interpreted in the same way as the coefficients obtained through an analysis of multiple lineal regression. Furthermore, it tested if the path coefficients were significant through the bootstrapping method from the value obtained through t-Student. Finally, the residual mean square root normalisation coefficient was tested, considering it an adequate fit when the values are less than 0.08 (Hu & Bentler, 1999).

## Results

### Internal consistency

In Table 2 appear the factorial loads of the items, as well as the composite reliability of each resultant factor and their Cronbach alfa coefficients. Taking into account the thresholds and recommended statistical values, the items that never surpassed the threshold were eliminated (D1_1; D1_2; D1_8; D2_9; D3_29; D5_22; D6_36; D7_14; D7_17; D7_18: D7_19).

**Table 2:** *Internal consistency reliability and Composite Reliability*

Dimension	Item	Loading	Composite Reliability	Cronbach’s alpha of each factor
D1: digital skills to search for information, manage it, analyze it and communicate results	D1_3	0.783	0.885	0.840
	D1_4	0.791		
	D1_5	0.800		
	D1_6	0.800		
	D1_7	0.720		
D2: digital ethics in digital research	D2_10	0.851	0.939	0.917
	D2_11	0.932		
	D2_12	0.875		
	D2_13	0.907		
D3: digital flow in research work	D3_26	0.751	0.872	0.779
	D3_27	0.871		
	D3_28	0.874		
D4: anxiety towards the use of ICT resources for research	D4_30	0.894	0.930	0.908
	D4_31	0.806		
	D4_32	0.808		
	D4_33	0.897		
	D4_34	0.857		
D5: quality of research-related ICT resources	D5_23	0.817	0.877	0.791
	D5_24	0.868		
	D5_25	0.832		
D6: intention to use ICTs for research work	D6_35	0.752	0.909	0.874
	D6_37	0.885		
	D6_38	0.902		
	D6_39	0.808		
	D6_40	0.725		
D7: Integration ICT resources for research	D7_15	0.719	0.860	0.783
	D7_16	0.768		
	D7_20	0.740		
	D7_21	0.879		

### Convergent validity

Table 3 presents the AVE coefficients for the factors of the instrument. As can be seen, the obtained values for each factor are greater than 0.50, with which it is established that more than 50% of the variance in the teachers score in the instrument is due to their indicators. As such, the AVE coefficients for the model factors grant an appropriate level of convergent validity that varied between 0.697 and 0.974.

**Table 3:** *Convergent validity*

	Average variance extracted (AVE)
Anxiety	0.728
Ethics	0.794
Flow	0.696
Integration	0.607
Intention	0.668
Quality	0.704
Skills	0.608

### Discriminant validity

the discriminant value was checked using the Fornell-Larcker criteria, showing the extent to which one factor is different from the other factors of the instrument, just as for the HTMT ratio. Table 4 shows the first analysed criteria, where the coefficients highlighted in grey (square root of AVE) is greater than the values produced below the diagonal line.

**Table 4:** *Fornell-Larcker criterion*

	Anxiety	Ethics	Flow	Integration	Intention	Quality	Skills
Anxiety	0.854						
Ethics	-0.586	0.891					
Flow	0.226	-0.203	0.834				
Integration	-0.026	0.156	0.514	0.779			
Intention	-0.117	0.151	0.494	0.459	0.817		
Quality	0.012	-0.026	0.243	0.202	0.098	0.839	
Skills	-0.501	-0.535	0.491	0.387	0.131	0.137	0.779

Table 5 shows the coefficients obtained for the second analysed criteria (grey background), obtaining values great than 0.85. Both criteria grant a second contact with an appropriate discriminate value of the proposed model.

**Table 5:** *Heterotrait-Monotrait Ratio (HTMT)*

	Anxiety	Ethics	Flow	Integration	Intention	Quality	Skills
Anxiety							
Ethics	0.631						
Flow	0.270	0.269					
Integration	0.069	0.172	0.627				
Intention	0.146	0.172	0.596	0.548			
Quality	0.070	0.072	0.310	0.245	0.126		
Skills	0.564	0.641	0.586	0.429	0.178	0.162	

On the other hand, said validity was also tested through analysis of the crossed loads, analysing how the items of one underlying factor correlate with those of other underlying factors, in order to ensure that the item is significantly in the appropriate factor over the rest. In Table 6 the items that correspond to their underlying factor are highlighted in order to distinguish them from the rest, demonstrating the strong correlation that it has with its corresponding factor and the weak correlation with the rest.

**Table 6:** *Cross Loadings*

	Anxiety	Ethics	Flow	Integration	Intention	Quality	Skills
D1_3	0.385	-0.332	0.524	0.446	0.136	0.150	0.783
D1_4	0.470	-0.641	0.283	0.093	-0.043	0.068	0.791
D1_5	0.374	-0.356	0.355	0.271	0.069	0.133	0.800
D1_6	0.346	-0.373	0.400	0.403	0.224	0.095	0.800
D1_7	0.399	-0.451	0.281	0.202	0.079	0.067	0.720
D2_10	-0.503	0.851	-0.234	0.083	0.104	-0.030	-0.504
D2_11	-0.538	0.932	-0.166	0.179	0.133	-0.014	-0.466
D2_12	-0.547	0.875	-0.217	0.092	0.140	-0.038	-0.515
D2_13	-0.518	0.907	-0.155	0.155	0.153	-0.022	-0.469
D3_26	0.319	-0.352	0.751	0.262	0.409	0.234	0.444
D3_27	0.143	-0.060	0.871	0.569	0.387	0.208	0.436
D3_28	0.120	-0.124	0.874	0.426	0.447	0.168	0.349
D4_30	0.894	-0.509	0.248	0.039	-0.084	0.044	0.507
D4_31	0.806	-0.388	0.120	-0.027	-0.129	0.044	0.329
D4_32	0.808	-0.373	0.158	-0.040	-0.100	0.003	0.313
D4_33	0.897	-0.561	0.148	-0.037	-0.156	-0.005	0.480
D4_34	0.857	-0.618	0.269	-0.058	-0.039	-0.031	0.455
D5_23	-0.048	0.038	0.194	0.226	0.126	0.817	0.091
D5_24	0.008	-0.066	0.206	0.103	0.061	0.868	0.110
D5_25	0.076	-0.050	0.210	0.161	0.052	0.832	0.145
D6_35	-0.080	0.110	0.395	0.318	0.752	0.130	0.091
D6_37	-0.050	0.115	0.485	0.418	0.885	0.086	0.152
D6_38	-0.081	0.119	0.452	0.385	0.902	0.105	0.115
D6_39	-0.088	0.083	0.381	0.445	0.808	0.071	0.164
D6_40	-0.212	0.209	0.280	0.290	0.725	-0.002	-0.019
D7_15	-0.050	0.120	0.350	0.719	0.450	0.157	0.324
D7_16	-0.058	0.119	0.334	0.768	0.324	0.122	0.238
D7_20	-0.032	0.146	0.326	0.740	0.300	0.161	0.186
D7_21	0.039	0.109	0.547	0.879	0.347	0.183	0.410

### Evaluation of the structural model

For the purpose of knowing if the relation between the underlying factors is significant, answers were required to be given to the following question: (1) What percentage of the variance of the endogenous variables is explained by the rest of the exogenous variables of the proposed model? and (2) To what extent do the exogenous variables contribute to predicting the explained variance of the endogenous variables? In order to answer the first question, the determination coefficient (R^2^) is used, while for the second question the path coefficients are used between the casual relations between such variables.

**Fig. 2:**
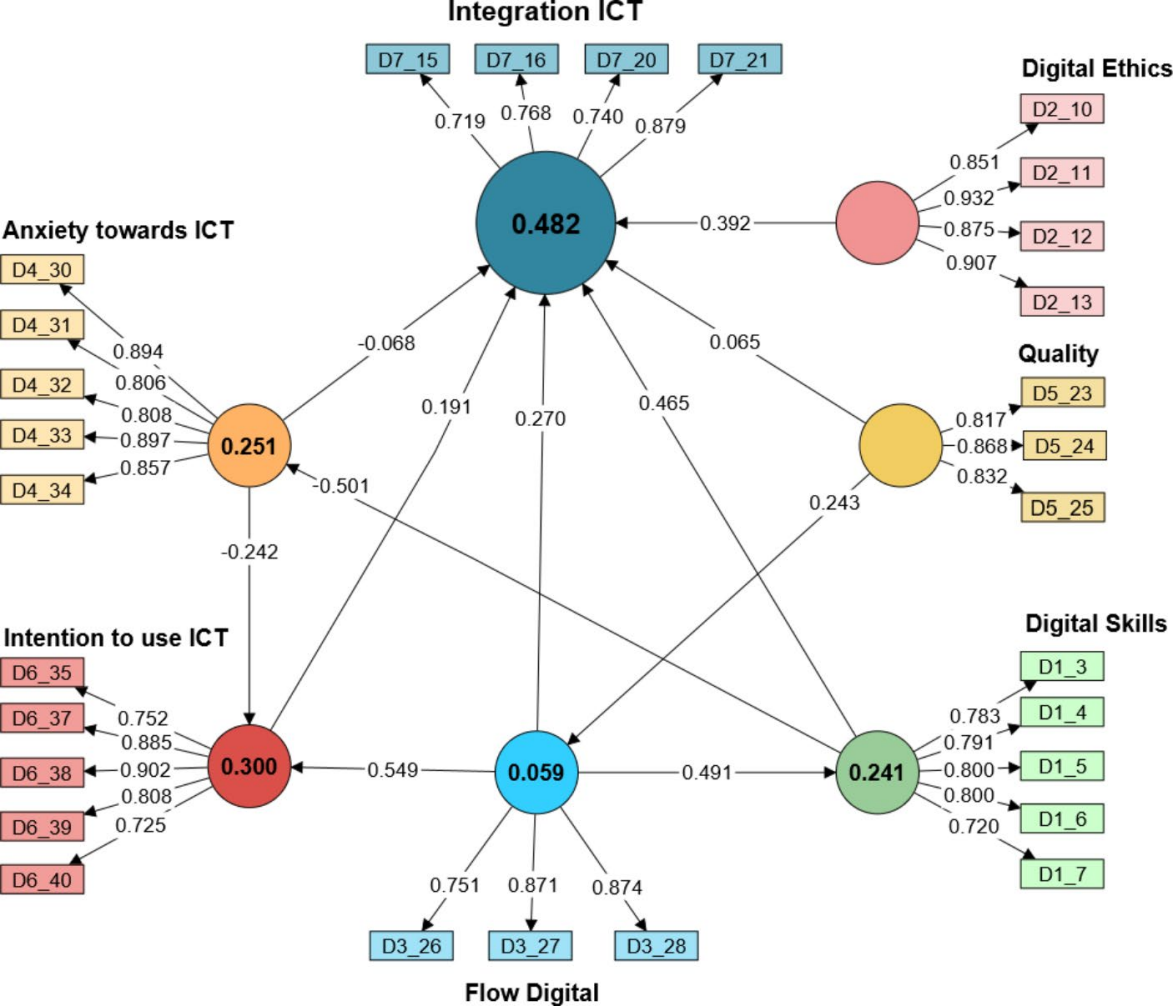
The Hypothesized Structural Results

Figure 2 observes that the underlying factors included in the model explain 48.20% of the integration variable variance; the 30% of the intention factor variance is explained by factors anxiety and flow; the 24.10% of the abilities factor variance is explained by the flow factor; the 25.10% of the anxiety variable variance is explained by the ability factor; and finally, the quality factor explains the 5.90% of the flow factor variance.

Table 7 shows the path weights in the established hypothesis, the level of significance between such relations and their corresponding effect sizes. Hypothesis 1 (H1) determines whether the behavioral intention of the researcher regarding the use of ICT in the research process significantly affects the subsequent integration in the research process. The link between the two factors is significant, reporting a t-value of 8.534 (β = 0.191, p-value < 0.01). Therefore, H1 is accepted. The hypotheses 2 (H2) about the relationship between the researcher’s digital skills and their subsequent integration into the investigative process (β = 0.465, t-value = 6.642, p < .001), is also corroborated. The hypotheses 3 (H3) focus on whether the researcher’s digital skills in the use of specific digital resources specific to the research area have a significant relationship with the level of anxiety that they can feel when using them. Results confirm this hypothesis (β = − 0.501, t-value = 25.366, p < .001).

Hypothesis 4 and 5 (H4 & H5) focus on whether the researcher’s flow state on using digital resources in research tasks has a significant relationship with, firstly, the behavioral intention of using these resources in the research process, to later integrate them into this process. Results confirm the two hypotheses: H4 (β = 0.549, t-value = 25.606, p < .001), H5 (β = 0.270, t-value = 9.092, p < .001). In addition, it was also confirmed how the state of digital flow significantly affects to the development of the digital skills of the researcher, hypothesis H6 (β = 0.491, t-value = 26.000, p < .001).

Hypothesis 7 (H7) tested whether the researcher’s state of anxiety about the use of specific digital resources used in the research process has an impact on the behavioral intention to use these resources, and later on the integration itself in the research process (Hypothesis H8). Results showed that teacher anxiety significantly negative effects on the behavioral intention to use ICT in research work (β = − 0.242, t-value = 11.627, p < .001), and its subsequent integration, albeit with minimal incidence (β = − 0.068, t-value = 2.437, p < .001). H7 and H8 are supported.

Hypothesis 9 (H9) hypothesized that digital ethical standards had a significant effect on the integration of ICT resources in the research process. The PLS-SEM findings revealed that the use of digital ethical norms had a significant prediction in the integration of ICT in this process (β = 0.392, t-value = 15.268, p < .005), which argues for H9. Lastly, the significant relationships between the quality of the technological resources and the state of flow of the researcher (Hypothesis H10) (β = 0.243, t-value = 9.403, p < .001) and their subsequent integration in the research process (Hypothesis H11) (β = 0.065, t-value = 3.609, p < .001), are also accepted.

**Table 7:** Summary of hypothesis testing

Hypothesis		Path coefficient (β)	t-value	*Significance (p-Value)*	Effect size (f^2^)
H_1_	Intention -> Integration	0.191	8.534	0.000**	0.08
H_2_	Skills -> Integration	0.465	16.642	0.000**	0.02
H_3_	Skills -> Anxiety	− 0.501	25.366	0.000**	0.05
H_4_	Flow -> Intention	0.549	25.606	0.000**	0.08
H_5_	Flow -> Integration	0.270	9.092	0.000**	0.03
H_6_	Flow -> Skills	0.491	26.000	0.000**	0.01
H_7_	Anxiety -> Intention	− 0.242	11.627	0.000**	0.10
H_8_	Anxiety -> Integration	− 0.068	2.437	0.015*	0.10
H_9_	Ethics -> Integration	0.392	15.268	0.000**	0.13
H_10_	Quality -> Flow	0.243	9.403	0.000**	0.02
H_11_	Quality -> Integration	0.065	3.609	0.000**	0.05

Note: *p < .05, ** p < .01

As can be seen in Table 7, all of the established relations between the factors were significant. To assess the strength of the relationship between the factors, the effect size coefficient is used (f^2^). This was calculated using the f^2^ procedure proposed by Cohen (1988), where a value equal to or less than 0.02 is interpreted as a small effect, a value of 0.15 as a medium effect and a value of 0.35 as a large effect. Through the PLS- SEM calculation, the values of f^2^ ranged from 0.01 to 0.13, all of them with minor effects. Detailed information on the effect sizes for each pathway is shown in Table 7. On the other hand, the SRMR criteria produced a coefficient of 0.78, being less than the 0.8 value recommended by Hu and Bentler (1999).

## Discussion

The main purpose of this study was to investigate the impact of the integration of specific digital resources by Higher Education teachers in the research process. For this, a measurement instrument was prepared with those factors and their relationships which significantly affect digital integration. This instrument is built from the findings of other researchers on incident factors in teacher digital competence, specifying all this in a new validated and relevant instrument which is linked to digital competences specifically in the teacher’s research process.

The instrument was configured by a total of 29 items distributed between the 7 established dimensions: digital abilities to search for information, managing it, analysing it and communicating the results (5 items), digital ethics in digital investigation (4 items), digital flow in investigation works (3 items), anxiety towards the use of ICT resources for investigating (5 items), quality of the ICT resources used for investigation (3 items), intention to use ICT for investigation works (5 items), ICT resource integration for investigating (4 items). After eliminating those items that did not fit with the required psychometric criteria, it can be concluded that the reliability of the instrument presents very satisfactory coefficients in all of the dimensions. The data, likewise, show that the discriminant validity is appropriate for the proposed model (Bagozzi & Yi, 1988), just as the discriminant validity in all the analysed criteria (Henseler et al., 2015; Clark & Watson, 1995), showing the correct saturation of all the items in their corresponding dimensions.

The hypotheses established between the integration of ICT and its possible incident factors were verified. The findings revealed a significant influence path from factor integration to digital skills, ethics, flow digital, and behaviour intention. Regarding the first hypothesis (H1), a link was confirmed between Intention to use ICT by the teacher and integration of these digital resources in the research process, corroborating the relationships found by Kovalik et al. (2013) and Ndlovu et al. (2020). In other words, achieving a real use of technology in the teacher’s research work is based, among other aspects, on the intention to use it (Shiue, 2007). Knowing that intentions have the potential to predict future integration, a comprehensive understanding of this factor as future work could help universities prepare constructive plans to increase the teacher training in scientific competences (Lovat et al., 1995), under the umbrella of efficiency in ICT (Tanjung, 2019).

Our next hypothesis (H2), it was also confirmed that there is a link between Digital skills and integration of digital resources in the research process. The digital skills of teachers in the use of technological resources in research processes has the third largest impact compared to the rest of the factors of the causal model, which is consistent with our earlier findings of Alazam et al. (2013) and Teo (2009). This discovery indicates the importance of teacher training in their practical domain for the use of technological resources in the scientific process (Pérez & López, 1999; El Hassani, 2015; Murnane & Levy 1996). In addition, as stated by Revilla et al. (2017), the continuous use of digital skills by teachers is a key factor that will positively influence the decrease in teachers’ negative attitudes about their levels of stress and anxiety, reaffirming the link between the dimension’s skills and anxiety, found in hypothesis H3. These results invite to reflect on the importance of permanent teacher training by the institutions that hire the TRS (teaching and research staff) both to reduce their levels of reluctance regarding technology in research work, but what is more important, so that they integrate into their research those digital resources that help them generate scientific knowledge more quickly and effectively among members of the scientific community (Tanjung, 2019; Arcila-Calderón et al., 2015).

The fourth and fifth hypotheses of the causal model (H4 & H5) showed a link between the digital flow and the intention of use, as well as the digital flow and the integration of digital resources in the research process. The results showed how the researcher’s digital flow has the biggest impact on the intention to use technology in the research process, and later its real integration in this process, coinciding with the results of Kim and Jang (2015), Calvo-Porral et al. (2017) and Rodriguez-Sanchez et al. (2008). These results underscore the need to encourage teachers to enjoy and be interested in technology for their scientific productions, either with incentives or reductions in teaching hours to be able to research, since, as Huang and Liao (2017) confirm, if the researchers are fully involved and concentrated in this process, they can even forget about time, fatigue and everything else, except the activity itself (Nakamura & Csikszentmihalyi, 2009), in our case, the improvement of research processes in the digital age (López-Martín et al., 2017; Mandal, 2018). In addition, it was verified that there was a link between the teacher’s digital flow and their digital skills in the research process, confirming the hypothesis H6. That is to say, if the researcher has a good state of digital flow, their digital skills will also be better and consequently it will have an impact on the integration of the research process.

The findings also corroborate the hypotheses H7 and H8, confirmed that there is a link between anxiety towards technology and the intention to use digital resources for research (Babie et al., 2016; Knezek & Christensen, 2016; Ünal et al., 2019), and consequently towards the integration of digital resources by the teacher in the research process (Joo et al., 2018; Paraskeva et al., 2008). However, the results have shown a much greater inverse relationship on the intention than on the integration itself. A plausible explanation for these findings is that teachers could have a high level of stress towards the intention to use digital resources for research, but as they integrate it into their research tasks, little by little these levels disappear.

In relation to hypothesis H9, it is observed highlights a link between the digital ethical standards and the integration of these resources in the research process (H9). Although it is observed that this factor has a good significant weight in the integration of digital resources, few answers exist today about the causal relationship between these factors. These findings should invite reflection and seek possible answers that help to better explain this relation. What is clear, as stated by Mbunge et al. (2021) is that an ethical and digital framework is needed to use technology in the best possible conditions.

Lastly, the last hypotheses (H10) evidenced a link between the quality of technological resources and the digital flow (the enjoyment and motivation of teachers in their scientific processes), since the enjoyment experience can be greater if accessibility to technology is adequate (Lin et al., 2012; Gil-Flores et al., 2017), along with technical and training support (Lawrence & Tar, 2018). The model presented also highlights the relationship between the quality of technological resources and the integration (H11). This relationship is characterized by statistical significance; however, the incidence (β) is not highly significant for integration development. Although authors such as Gil-Flores et al. (2017) have determined that the access, availability, and quality of digital resources could affect their integration into the educational process, it must be considered that “teachers are reluctant to use technology as a teaching tool if the tool is not good” (Shiue, 2007; p. 446). Therefore, a plausible explanation of these findings is since the study has been carried out in a developed country which is committed to the advancement of innovation in technological matters through subsidies to university institutions. However, these statements must be taken with caution and the plausible causes of their low incidence in integration by teachers continue to be analyzed.

## Conclusions and future works

Nowadays it is fundamental to have the tools to measure the level of development of university teachers’ digital competency to carry out investigation work, seen as it is fundamental that such teachers contribute to the building of knowledge and social transformation (Rubio et al., 2018; George & Salado, 2019; Suárez-Triana et al., 2020), being it essential that there exists a solid scientific community (Arcila-Calderón et al., 2015) and strongly interlinked.

The main results of this study showed that there was a direct and significant effect between the six factors analyzed in the causal model and the integration of digital resources in the research process by the Higher Education teacher. It has been found that the factors with the greatest incidence in the integration by the teacher have been the digital flow and the digital skills of the researcher. Therefore, a good implication in practice, not only for teacher-researchers and their professional development, but also for novice researchers and classroom teachers who can carry out small experiments with their group-class, is to emphasize training courses. MOOCs could be useful and be the basis for teacher training, motivating them with strategies on how to apply these resources in their educational processes.

However, other causal factors of the causal model, such as teacher anxiety about the integration of technological resources in the research process, have not had a great impact on teacher integration, although it has been a greater weight on the intention factor. These results invite us to continue looking for answers about why it affects one factor more than another. As future work, it would be interesting to know in depth the psychological state of teachers regarding the use of specific technological resources in their research process, which can generate anxiety and/or push them to adopt skeptical attitudes regarding the use of digital resources.

Although the factor with the least incidence on the integration of digital resources in the research process has been the researcher’s perception of the quality of the technological resources available to them, there may be considerable disparities between developed and developing countries. Educational institutions in developed countries tend to have more grants and technological resources. It would be important to demonstrate the value of the quality of resources and technological infrastructure that university institutions offer teachers for research. In this way, we could think about how this affects, to a greater or lesser extent, its integration in the research process.

In addition to reflecting and concluding on the results of the incident factors in the integration of technology in the research process, we now have to reflect on how to improve the design and methodology of the study. A weakness of the study is the type of sample used since it was non-probabilistic. Therefore, the results obtained must be interpreted with caution to other teachers with similar characteristics and not extrapolate the findings to all researchers. For this reason, it would be interesting as future work to be able to collect a more representative sample of researchers with the purpose of being able to generalize the results and that the instrument is equally valid for the entire scientific community.

In short, an effective researcher in the digital age will be able to consolidate an adequate professional identity not only with specialized knowledge in his area of knowledge, but also with instrumental skills for research activity on the Internet. This digital competence of the researcher is not reduced only to the knowledge and use of the necessary skills for the management of digital resources in the scientific process, but this competence has must also encompasses other factors such as motivational and enjoyment towards technology (flow), quality of resources technology, as well as ethical and behavioral attitudes about the intention to use of technology. A good researcher in the information and communication society must have good levels in all these incident factors in order to be able to develop professionally in a digital environment, since it is precisely through digital media that the rest of the scientific community is aware of its members.

## Data Availability

Not applicable.
